# The Proper Use of Ergot in Obstetrical Practice*Read before the Buffalo Obstetrical Society, April 27, 1886.

**Published:** 1886-09

**Authors:** Frank Hamilton Potter

**Affiliations:** Assistant to the Chair of Therapeutics and Materia Medica, Niagara University; 284 Franklin Street


### *THE PROPER USE OF ERGOT IN OBSTETRICAL PRACTICE.**

By FRANK HAMILTON POTTER, M. D.,

Assistant to the Chair of Therapeutics and Materia Medica, Niagara University.

If the diversity of opinion, regarding the action of a medicine, may be taken as the measure of our ignorance concerning it, we

*Read before the Buffalo Obstetrical Society, April 27, 1886.

can safely say that the virtues of ergot are yet to be discovered. It has been advised for the most various and opposite conditions and the literature of the subject has grown to such an enormous extent that it would take an entire evening to simply mention what has been written upon it. If, however, we examine this mass of conflicting statements carefully, we will readily discover that most authorities are agreed upon certain of its physiological properties, and so it would not be very rash to advance the opinion that it should only be used along the lines thus indicated. Therefore, before considering whether it has, or not, a place in the armamentarium of the obstetrician, it may be well to state briefly its physiological actions. It is an adventitious product belonging to the vegetable kingdom, but wanting in one of the marked characteristics of the latter, in that it contains no chlorophyl. It is a fungus and lives by feeding upon a higher form of life. It is, therefore, a parasite. It is gathered in that state of suspended animation which some fungi exhibit between the mycelial stage and the development of the thallus. It is a very complex substance containing many bodies peculiar to itself, besides a certain per centage of a fixed oil. It may be used in the form of powder, a fluid extract or an aqueous extract, but as preparations are prone to a more or less rapid deterioration they must be carefully selected and used fresh. Many conflicting statements concerning the action of ergot can be explained by the changes it has undergone between the times of its manufacture and employment. This is an important subject and bears directly upon the use of many drugs besides the one under consideration. As far as ergot is concerned it is only necessary to state Dr. Squibb's conclusions in regard to it, to indicate the great importance of using a pure and fresh sample of the drug. He says: "The requisite of any trustworthy preparation of ergot is, of course, a uniform good quality in the drug, and this is, by no means, easy to obtain. The market is overstocked with ergot, at extremely low prices. Tons of it might be had at sixteen to eighteen cents per pound, but it is all so small that it is almost certain that it is from oats, barley, or wheat, rather than from rye; while its deficiency in odor and taste, and its uncleanliness forbid the idea of its being trustworthy. Most of it is imported in bags, and thus only by chance can arrive in proper conditions. Much of it is contaminated with seeds of various weeds, and requires much labor in cleaning, as the weed seeds are often bitter, and sometimes poisonous. It is not unfrequently wormy, or bears the marks of having been cleaned from worms and worm-dust, to improve the chances of sales and profits. Occasionally lots, and these often large lots, look as if they had been washed, and suggest the idea of having been partially exhausted for the making of the so-called ergotins. Hence, it may be easily understood that to obtain a uniform supply of good ergot is very difficult, even with the screw of prices entirely taken off." In another place, he says: "The molecular constitution of the active portion of the drug seems, however, in its natural condition, to be loose, and, like a slow fermentation, to be undergoing slow molecular changes, so that, by age, its peculiar activity is slowly diminished, until finally lost." The ergot in the grain, however well kept, is known to become inactive, without any known change in appearance, though the sensible properties, such as odor and taste, may be, and probably do change.

Ergot in powder is known to diminish in activity much more rapidly than in grain, and probably soon become inert. The tincture and wine of ergot are believed to change, though more slowly than the ergot in substance, whilst the extracts and so-called ergotins are all supposed to change more rapidly.

From this it can be seen that there must be many useless preparations on the market, and that great care is necessary in order to obtain a good specimen. Having then obtained a pure sample, what actions are you to expect from it? I refer, of course, to its action from medicinal doses, its toxic effects being left out of consideration. It is a heart depressant, slowing its action and rendering the contractions less vigorous. The blood pressure, therefore, at first, falls. This, however, is soon overcome by its action on the nervous system, and then the blood pressure rises. It is a stimulant to the centric nervous system, causing a marked contraction of the blood vessels, which, as has just been said, more than counterbalances the depressant effect upon the heart. Boldt also declares that through the contracted capillaries can be observed to run wave-like peristallic spasms. I have seen no confirmation of Boldt's statements, though it can readily be seen that, if true, they would have an important bearing on our conclusions. It acts also upon the involuntary non-striated muscular fibre wherever found, causing it to contract. These contractions are tonic and not intermittent. It causes tonic contractions of the enlarged uterus—I say enlarged uterus, for it makes no difference whether the enlargement takes place through the growth of an ovum, or the growth of a tumor. After the muscular fibre has been developed to a certain point, it becomes susceptible to the action of ergot. Before this no effect is observed, and this explains the influence of the drug over the womb, in the latter months of pregnancy, while in the earlier months no influence at all is observed. It is a true oxytocic; though the effect it produces is very far from being that observed when the uterus contracts naturally; and a study of the differences between the normal action of the uterus and the action produced by the exhibition of ergot, will indicate whether it should be used, or not, during labor, for its peculiar effects.

When the hand is placed upon the abdomen, over the pregnant uterus, and allowed to remain there for a few moments, the tumor beneath will be observed to grow hard and tense, and then again to relax and become soft. How early in pregnancy this condition of affairs exists, I do not know, but probably as soon as the uterus emerges from the pelvic cavity. This rythmical contraction and relaxation is considered by some writers, especially by Lusk and Lawson Tait, as one of the earliest and most reliable signs of pregnancy, no other tumor in the abdomen, or elsewhere, ever exhibiting these phenomena. As the pregnancy draws to a close these contractions grow stronger and stronger and follow each other with ever-increasing rapidity until the womb is emptied of its contents. There is no reason to think that the "pains" of labor differ, excepting in degree and frequency, from the unconscious contractions discovered only where the hand is pressed closely upon the abdomen of a pregnant woman. These contractions never become tonic but are always alternating, or if, for any reason, the womb does contract tonically, the normal physiological relations no longer exist, and the condition must be considered pathological. This rythmical movement of the uterus is necessary to preserve the life of the fœtus. During the contractions the contents of the womb are pressed upon from all sides, and the placental circulation seriously interfered with, if not entirely obliterated. A prolongation of this condition, would of course, cause the death of the fœtus through asphyxia. Other interesting phenomena attend a normal labor. The contractile wave begins at the fundus and extends gradually to the cervix, at the same time the circular fibres surrounding the cervix retract, thus causing the os to dilate. If now, for any reason, this process is interfered with, and retraction and dilation prevented, the womb becomes unable to expel its contents, and the destruction of the child results. All this time the glands in the female genitalia, are secreting copiously and rendering the maternal soft parts pliable and dilatable. Whatever other attendant phenomena there may be, sufficient has been mentioned to show the complicated nature of the process and to indicate what a drug should do in order to be an assistance to delivery. It should not interfere with the regular rythmical contractions of the uterus; it should not interfere with the dilatation of the os; it should not interfere with glandular action, thereby rendering the soft parts dry and unyielding. What now, have we learned concerning the action of ergot? It causes tonic and persistent contractions of the womb; it locks up the secretions; it prevents softening and dilatation of the os uteri, and the surrounding soft parts, it fails not only to render assistance to a single one of the physiological indications of labor, but, on the other hand, puts obstacles in the way, which are often serious, and prevent a successful termination of parturition.

These conclusions, which have been reached from a study of the physiological action of the drug, will be sustained, I think, by a review of its clinical history. Long ago Dr. Emmet warned the profession of the great danger of continued pressure, unrelieved by the recession of the child's head, in lingering or delayed labor. By this delay the soft parts of both mother and child are liable to become necrotic, through the interference with the circulation, and, more or less, complete destruction follow, according to the length of time the pressure is kept up. There may result vaginitis, pelvic cellulitis, or sloughing, of a more or less extensive surface. The destruction may be so slight as not to be readily detected, or so great as to seriously endanger life. On the other hand, cases have been recorded where the stimulation to uterine contraction has been so powerful, probably through a peculiar susceptibility to the action of the drug, that the child has been at once thrust into the world, in spite of the resistance of the undilated soft parts, and all the lacerations occuring singly, or in groups, from rupture of the uterus, to rupture of the perineum, have followed. Again, it may act upon the entire uterus, causing a powerful spasm, as has just been mentioned, or upon a special portion of it, causing irregular contractions. The middle portion may be effected, resulting in hour-glass contraction, or the circular fibres around the cervix, preventing retraction and dilatation, or the fundus alone, or a single cornus, causing irregular and ill-directed efforts at expulsion. This uncertainty in the action of ergot, even where it is pure, should, in the absence of all other considerations, render us very cautious in its use. What should be done in these cases of lingering or delayed labor does not fall within the scope of this paper to discuss. The various methods of treatment are familiar to all. I merely wish to point out that ergot is not the proper means to employ, on account of the dangers attending its use, and the uncertanity of its action. Besides the necroses and lacerations, already referred to, the life of the child may be endangered by the use of ergot. Many years ago Dr. Beatty, of Dublin, and Dr. Eve, of this country, called attention to the danger resulting from the absorption of the essential oil of ergot, claiming that it acted as a poison upon the fœtus, destroying its life, even when there was no visible effects upon the uterus. Since that time this subject has been thoroughly investigated, and still-birth is now recognized as one of the deplorable results that may follow the use of this drug. This may result from the poisonous action of its essential oil upon the fœtus, or from the compression of the latter, and the interference with the placental circulation, when, after its use, the womb is thrown into a state of tonic spasm, but more likely from a combination of both these causes. Meigs has declared that " multitudes of children were dead-born from this cause, by the exhibition of a medicine, which, as certainly excites spasm of the womb as nux vomica does of the other muscles of the body." In 1850 the commission appointed by the Academy of Medicine, of Paris, to investigate the influence of ergot upon the life of a child, reported " its life endangered both by compression and narcotism." And three years afterwards the Academy formally adopted the conclusions of M. Depaul, that " except in miscarriage, in certain labors, attended with hæmorrhage, and, occasionally, at the conclusion of natural labor, parturient women would be gainers by the complete disuse of ergot." I learn, also, from Dr. Johnson, of Washington, who read a paper on this subject before the American Gynæcological Society, in 1882, that Dr. Stearns, who re-introduced this remedy to the profession, and who used it more cautiously than many do at present, " lost his practice from a strange mortality among the children, and from child-bed fever, which followed him like an evil genius from door to door."

Prof. Chapmann, of the Long Island College Hospital, has stated that " there can scarcely be any doubt that one hour's ergot-pain would be fatal to one-half the children born, for they are literally smothered, since they are effectually shut out from the very source whence their blood can be supplied with oxygen." And again, that " we should never use ergot as an expulsive agent, never where the pains have been, and are vigorous, never to overcome impediments, such as narrow pelvis or rigid perineum, and never in primipara, under any circumstances." This kind of evidence could be multiplied until we would all be wearied with its recital, but sufficient has been said to show the prevailing opinion among the teachers upon this subject. I am inclined to think, however, that these words of warning are not heeded, and ergot is still used too frequently by many physicians.

This drug has been used also in cases of threatened or actual abortion,and retained placenta and membranes. But from its action it will be readily seen that instead of assisting to expel the ovum, or the membranes, it really shuts them up in the uterus, and prevents the employment of far safer means. It may possibly become necessary to remove the ovum, the retained plancenta, or membranes, and by the use of ergot you have rendered the os uteri rigid and unyielding, which increases, ten-fold the difficulty of so doing. Besides, by shutting up material which no longer has a physiological relation with the uterus, and is prone to decomposition, you may, through absorption of this material, find yourself confronted by a dangerous and insidious form of puerperal septicæmia. On the whole, then, the dangers of its employment are too great, and it is safe to say that ergot should never be used in this class of cases.

It has been advised also as a means of preventing and curing post-partum hæmorrhage, and it would seem a priori to be especially adapted to this class of cases. Its proper use, however, depends largely on the cause of the hæmorrhage and the time when it takes place. If the hæmorrhage occur before the birth of the placenta, it is the best practice to remove the latter at once, and then cause the uterus to contract by the application of astringents to its inner surface, at the same time kneading it externally, ergot should then be administered, hypodermically with the hope of its preventing a relaxation of the womb and a recurrence of the bleeding. The danger of shutting up the placenta in the uterine cavity has already been mentioned, and in order to avoid the unfortunate complications resulting from this accident, the administration of ergot should be deferred until the womb is entirely empty.

There are other cases in which, after the womb has once firmly contracted, secondary relaxation occurs, followed by hemorrhage. Here ergot can be used with a great benefit, as tonic contraction of the uterus is just what is most desired. The literature is full of cases of this description, in which ergot has been of great assistance, and even in some instances has been considered the sole cause of the successful results. This is its proper place as far as obstetrical practice is concerned, and I cannot help thinking that many of the dangers of the lying-in-chamber would be prevented if it were never used, excepting in those cases where, the uterus being empty, secondary relaxation threatened, or had actually occurred. Of course, it takes a few minutes for the drug to produce its effects, even when given hypodermically, and it should be supplemented by the other means usually employed in cases of this character.

In the preparation of this paper I have avoided, entirely, the reports of cases, the object being to present merely a connected outline of the subject, and to draw out from the members of the club their own experiences.

Nothing has been said of the use of ergot in the other departments of medicine, and this interesting subject must be reserved for some future meeting.

In conclusion, allow me to present the following points for consideration:

I. Ergot is a drug which in any of its preparations tends to deteriorate rapidly, and should never be used, excepting when prepared from a pure and fresh specimen.

II. It is a stimulant to the tubular and non-striated muscular structures of the body, causing them to contract.

III. It acts especially upon the muscular structure of the uterus, throwing it into a state of tonic spasm.

IV. Its action on the uterus is, however, uncertain; sometimes it contracts the entire organ, at others only a small part of it.

V. If the entire organ is contracted, labor may be delayed through the rigidity of the os, and the child destroyed by the interference of the placental circulation.

VI. Or the contractions may be so powerful as to force the child at once into the world, causing any or all of the lacerations of the soft parts of the mother.

VII. The life of the child may be endangered, also, through absorption of the essential oil of ergot.

VIII. If given after the birth of the child, and before the expulsion of the placenta and membranes, it may prevent the removal of the latter, and thus be indirectly a cause of puerperal septicæmia.

IX. It may act in a similar manner in cases of abortion, actual or threatened, and cause a similar result.

X. The proper use of ergot in obstetrical practice is limited to those cases in which, after the expulsion of the placenta, the uterus refuses to contract, or having once contracted shows a tendency to secondary relaxation. Even in these cases, however, reliance should not be placed upon it alone, but its action should be supplemented by the other means used to provoke uterine contraction.

284 Franklin Street.

